# Lower iodine storage in the placenta is associated with gestational diabetes mellitus

**DOI:** 10.1186/s12916-021-01919-4

**Published:** 2021-02-19

**Authors:** Kristof Y. Neven, Bianca Cox, Charlotte Cosemans, Wilfried Gyselaers, Joris Penders, Michelle Plusquin, Harry A. Roels, Karen Vrijens, Ann Ruttens, Tim S. Nawrot

**Affiliations:** 1 Centre for Environmental Sciences, Hasselt University, Diepenbeek, Belgium; 2 Department of Obstetrics, East-Limburg Hospital, Genk, Belgium; 3 Laboratory of Clinical Biology, East-Limburg Hospital, Genk, Belgium; 4 Louvain Centre for Toxicology and Applied Pharmacology, Université catholique de Louvain, Brussels, Belgium; 5 Sciensano, SD Chemical and Physical Health Risks, Tervuren, Belgium; 6 Department of Public Health & Primary Care, Leuven University, Leuven, Belgium

**Keywords:** Neonates, Placenta, Iodine, ICP-MS, Gestational diabetes mellitus, Pregnancy, DOHaD

## Abstract

**Background:**

The micronutrient iodine is essential for a healthy intrauterine environment and is required for optimal fetal growth and neurodevelopment. Evidence linking urinary iodine concentrations, which mainly reflects short-term iodine intake, to gestational diabetes mellitus (GDM) is inconclusive. Although the placental concentrations would better reflect the long-term gestational iodine status, no studies to date have investigated the association between the placental iodine load and the risk at GDM. Moreover, evidence is lacking whether placental iodine could play a role in biomarkers of insulin resistance and β-cell activity.

**Methods:**

We assessed the incidence of GDM between weeks 24 and 28 of gestation for 471 mother-neonate pairs from the ENVIR*ON*AGE birth cohort. In placentas, we determined the iodine concentrations. In maternal and cord blood, we measured the insulin concentrations, the Homeostasis Model Assessment (HOMA) for insulin resistance (IR) index, and β-cell activity. Logistic regression was used to estimate the odds ratios (OR) of GDM, and the population attributable factor (PAF) was calculated. Generalized linear models estimated the changes in insulin, HOMA-IR, and β-cell activity for a 5 μg/kg increase in placental iodine.

**Results:**

Higher placental iodine concentrations decreased the risk at GDM (OR = 0.82; 95%CI 0.72 to 0.93; *p* = 0.003). According to the PAF, 54.2% (95%CI 11.4 to 82.3%; *p* = 0.0006) of the GDM cases could be prevented if the mothers of the lowest tertile of placental iodine would have placental iodine levels as those belonging to the highest tertile. In cord blood, the plasma insulin concentration was inversely associated with the placental iodine load (*β* = − 4.8%; 95%CI − 8.9 to − 0.6%; *p* = 0.026).

**Conclusions:**

Higher concentrations of placental iodine are linked with a lower incidence of GDM. Moreover, a lower placental iodine load is associated with an altered plasma insulin concentration, HOMA-IR index, and β-cell activity. These findings postulate that a mild-to-moderate iodine deficiency could be linked with subclinical and early-onset alterations in the normal insulin homeostasis in healthy pregnant women. Nevertheless, the functional link between gestational iodine status and GDM warrants further research.

**Supplementary Information:**

The online version contains supplementary material available at 10.1186/s12916-021-01919-4.

## Background

Gestational diabetes mellitus (GDM) is a disease that is characterized by glucose intolerance with onset during the pregnancy and which attenuates after delivery [1]. Generally, 5% of all pregnancies are complicated by GDM [2], adversely affecting the intrauterine environment, which may in turn lead to congenital abnormalities and even spontaneous abortions [3]. Postpartum complications include a 7.5-fold increased risk at type 2 diabetes mellitus (T2DM) for the mothers [4] and a higher risk at obesity [5] and diabetes mellitus [5] for the offspring. There are well-established GDM risk factors such as a high body mass index (BMI), a family history of diabetes, and certain ethnic backgrounds [6]. More recently, low thyroid hormone levels during gestation have been postulated as an additional risk factor for GDM [7, 8].

The thyroid hormones play a vital role in fetal growth and neurological development [9]. To produce adequate quantities of these hormones, the body requires a sufficient iodine intake, which, according to the World Health Organization (WHO), corresponds to a minimum daily consumption of 250 μg of iodine for pregnant and lactating women [10]. Iodine deficiencies in adults, assessed using urinary iodine concentrations, have been linked with altered insulin and glucose homeostasis [11], mainly via the thyroid hormones [7]. For example, these hormones can reduce the availability of insulin by lowering the half-life of and increasing the breakdown of insulin [12]. Secondly, the expression of glucose transporter 2 in liver cells is increased, resulting in a higher hepatic glucose efflux [12]. In 2013, a nation-wide study in Belgium revealed that iodine intake in women of childbearing age and those in early pregnancy have an inadequate iodine intake [13], resulting in a mild-to-moderate iodine-deficient population. These findings were corroborated within the ongoing ENVironmental Influence *ON* early AGEing (ENVIRONAGE) birth cohort [14].

Considering the placental iodine concentration as a proxy of long-term iodine intake [15], to our knowledge, no epidemiological studies to date have investigated the association between gestational diabetes mellitus and placental iodine load. We postulate that placental iodine concentrations are linked with the incidence of GDM. Moreover, evidence is lacking whether placental iodine could play a role in the insulin homeostasis, insulin resistance, and β-cell activity.

## Research design and methods

### Research design

Mother-newborn pairs were enrolled in the ENVIR*ON*AGE birth cohort. The Ethics Committee of Hasselt University and East-Limburg Hospital (Genk, Belgium) approved the study protocol, which was carried out following the Declaration of Helsinki. All participating mothers were asked to provide written informed consent at the birth of their newborn in the East-Limburg Hospital. Mothers could be included in the cohort if they were able to fill out questionnaires in Dutch, did not have a planned cesarean, and provided written informed consent. Full details of the study protocol were published previously [16].

Questionnaires were used to collect sociodemographic information such as maternal birthdate, education, smoking status, pre-pregnancy body mass index (BMI), alcohol consumption during pregnancy, newborns’ ethnicity, and parity. We coded maternal education as “low” (no diploma or primary school), “middle” (high school), and “high” (college or university degree). Smoking status was classified as “non-smokers,” “cessation before pregnancy,” and “smoked during pregnancy.” Alcoholic beverage consumption was categorized as “none” or as “maximally one glass per day.” Ethnicity was coded based on the native country of the neonate’s grandparents as either “European” (at least two grandparents were European) or “non-European” (at least three grandparents were of non-European origin). For parity, the newborns were coded as primiparous (first child), secundiparous (second child), or multiparous (third or later child).

Medical records of the hospital were used to retrieve information on maternal weight gain during pregnancy, newborns’ sex, gestational age, and medical conditions such as GDM, thyroid problems, pre-eclampsia, and hypertension. GDM was determined between the 24th and 28th week of gestation at the hospital via the “two-step” approach of the American Diabetes Association [17]: a 50-g (non-fasting) glucose challenge test (GCT) followed by a 100-g oral glucose tolerance test (OGTT) if the GCT was positive. In short, mothers who had a glycemic value of more than 140 mg/dL or 7.8 mmol/L after 1 h during the GCT moved on to OGTT. During this test, they drank a solution containing 100 g of glucose, and the glycemic values were measured when sober (threshold 95 mg/dL or 5.3 mmol/L) and after 1 h (threshold 180 mg/dL or 10 mmol/L), 2 h (threshold 155 mg/dL or 8.6 mmol/L), and 3 h (threshold 140 mg/dL or 7.8 mmol/L). If at least two measurements were above the thresholds, the mother-to-be was diagnosed with GDM.

### Blood sampling, biochemical measurements, and HOMA-IR determination

We collected cord blood immediately after delivery in an 8 mL plastic BD Vacutainer^©^ Lithium Heparin tubes (BD, Franklin Lakes, NJ, USA) and maternal blood one day after delivery in plastic BD Vacutainer EDTA tube (BD, Franklin Lakes, NJ, USA). Samples were centrifuged at 2500*g* for 15 min, and the obtained plasma was frozen at − 80 °C. The clinical laboratory of the East-Limburg Hospital measured the plasma levels of insulin (pmol/L) and glucose (mmol/L) with an electro-chemiluminescence immunoassay using the Modular E170 automatic analyzer (Roche, Basel, Switzerland).

The insulin resistance (IR) and β-cell activity were assessed via the updated Homeostasis Model Assessment (HOMA) [18]. This technique calculates the β-cell function and IR from basal glucose and insulin concentrations. Therefore, it is necessary to provide clinically realistic values, which are representative for fasting subjects. Thus, plasma glucose should range between 3.5 and 25.0 mmol/L and plasma insulin concentrations between 20 and 400 pmol/L [18].

### Placental tissue collection and iodine analysis

Placentas were frozen at − 20 °C within 10 min after delivery. They were minimally thawed, and biopsies were taken at three standardized locations, situated at 2 cm from the umbilical cord. We cut away any membranes and rubbed the tissue against a Grade 54 filter paper (GE Healthcare, Chicago, USA) to remove any excess of blood. The tissue samples were stored in metal-free containers (Sterile propylene tubes; VWR, PA, USA) and were frozen at − 20 °C until iodine determination. For each placenta, equal amounts of the biopsies were pooled from each of the three sampling locations to obtain 500 mg of tissue that was placed in a 0.5% tetramethylammonium hydroxide (TMAH) solution and heated to 90 °C for 3 h in a DigiPREP block digestion system (SCP SCIENCE, Quebec, Canada). Iodine was measured via ICP-MS on an Elan DRC II (Perkin Elmer, MA, USA) on mass ^127^I, with ^125^Te as an internal standard. A more detailed description of the method is provided by Neven and colleagues [14].

### Statistical methods

Database management and statistical analysis were performed with the SAS software, version 9.4 (SAS Institute, Cary, NC, USA). Mean ± standard deviation (SD) is given for continuous variables and the proportion for categorical variables. The normality of the data distributions was tested with the Shapiro-Wilk statistic and quantile-quantile plots. To study the possible confounding structure in our data set, we assessed the distributions of continuous variables (ANOVA) and the proportions of categorical variables (*χ*^2^-statistics) across tertiles of the placental iodine concentrations.

In the first analysis, we assumed that placental iodine concentrations were causally linked to the likelihood of developing GDM. Therefore, we used a multivariate logistic regression model to evaluate the relationship between placental iodine load and the incidence of GDM. We accounted for the following a priori chosen variables based on previous findings [19]: maternal education, hypertension, smoking status, pre-pregnancy BMI, maternal age at delivery, gestational weight gain, alcohol use, neonatal parity, gestational age, ethnicity, and sex of the neonate. Correction for these variables was done in all the subsequent analyses as well. The results are presented as odds ratios (ORs) with the 95% confidence intervals (95%CI). To assess the public health significance of the GDM findings, we calculated the population attributable fraction (PAF) [20] for the total population and by contrasting the first versus the third tertile of placental iodine load.

In a second analysis, a generalized linear model was used to investigate the relationship of the maternal and cord plasma concentrations of insulin (collected at one day after delivery and at delivery, respectively) with the placental iodine concentrations in non-GDM mothers. In a sensitivity analysis, we included the GDM mothers (*n* = 12). Because the plasma insulin concentrations needed log-transformation to ensure normality, we presented these results as a percent change for a 5 μg/kg increment in placental iodine.

The HOMA-IR method [18] was used in a third analysis to assess the insulin resistance and β-cell activity in a healthy subset (i.e., excluding mothers with hypertension and/or GDM). We used a mathematical model of fasting plasma glucose and insulin concentrations to estimate insulin sensitivity.

### Role of the funding source

The funder of the study had no role in the study design, data collection, data analysis, data interpretation, or writing the report. The corresponding author had full access to all the data in the study and had final responsibility for the decision to submit for publication.

## Results

### Study populations

**Fig. 1:**
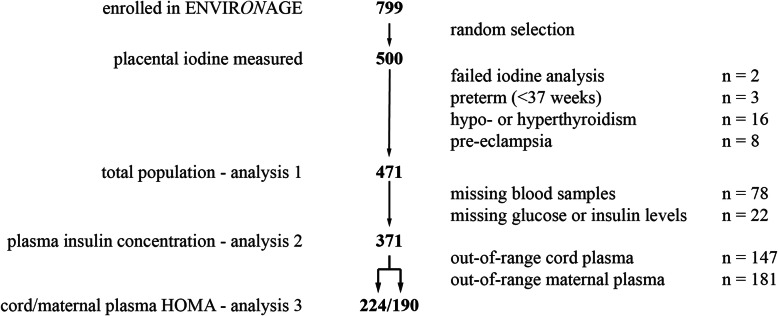
Flow chart depicting the selection procedure of the different study populations (analyses 1 to 3), selected from the ENVIR*ON*AGE birth cohort. For analysis 3, we excluded values that were out of range for the HOMA calculator: normal range for insulin is between 20 and 400 pmol/L and for glucose between 3.5 and 25.0 mmol/L

In the present study, we randomly selected 500 bio-banked placentas from 794 mother-neonate pairs who were enrolled in the ENVIR*ON*AGE birth cohort between March 1, 2013, and April 1, 2017 (Additional file 1: Table S1). We included 471 mother-neonate pairs in our first analysis after 29 exclusions: placental iodine below the limit of quantification (*n* = 2), preterm births (i.e., born before 37 weeks of pregnancy; *n* = 3), pre-eclamptic pregnancies (*n* = 8), and mothers with hyper- or hypothyroidism (*n* = 16). The second analysis involved insulin, for which we used a subset of only 371 pairs because of missing blood samples (*n* = 78) for either mother or child and glucose or insulin measurements (*n* = 22) due to coagulation or a limited amount of plasma. For the analysis concerning HOMA-IR, we excluded out-of-range values for plasma glucose (normal range 3.5 to 25.0 mmol/L) and plasma insulin (normal range 20 to 400 pmol/L) to obtain a final subset of 190 for mothers and 224 for neonates. Figure 1 provides a schematic overview of the study populations.

Table 1 presents the clinical and sociodemographic characteristics of the total population and the different subsets of mother-neonate pairs. In short, for the whole group, 20 mothers (4.3%) were diagnosed with GDM. Overall, the mothers aged on average 29.4 years (SD 4.4). The mean pre-pregnancy BMI was 24.6 kg/m^2^ (4.8), and most mothers never smoked (*n* = 303 [64.3%]) or consumed any alcohol during gestation (*n* = 407 [86.4%]). The mean weight of the neonates was 3462 g (424) at birth, and 242 (51.4%) were boys. The majority of the neonates had at least two European grandparents (*n* = 411 [87.3%]). The mean gestational age was 39.9 weeks (1.0). Over half of the neonates were primiparous (*n* = 249 [52.8%]) or secundiparous (*n* = 159 [33.8%]).

**Table 1:** Clinical and sociodemographic characteristics of the mother and neonate pairs for the total population and subsets

Characteristics	Total population (***n*** = 471)	Plasma insulin concentration (***n*** = 371)	Cord plasma HOMA (***n*** = 224)	Maternal plasma HOMA (***n*** = 190)
**Mother**				
Age, years	29.4 (4.4)	29.3 (4.3)	29.0 (4.3)	29.4 (4.3)
Pre-pregnancy BMI, kg/m^2^	24.6 (4.8)	24.2 (4.4)	24.9 (4.9)	24.6 (4.5)
Net weight gain, kg	13.9 (5.8)	14.2 (5.6)	14.5 (5.7)	14.3 (5.7)
Gestational diabetes mellitus				
*No*	451 (95.7%)	371 (100%)	224 (100%)	190 (100%)
*Yes*	20 (4.3%)	0 (0%)	0 (0%)	0 (0%)
Hypertension				
*No*	443 (94.1%)	350 (94.3%)	224 (100%)	190 (100%)
*Yes*	28 (5.9%)	21 (5.7%)	0 (0%)	0 (0%)
Self-reported tobacco use				
*Non-smoker*	303 (64.3%)	240 (64.7%)	144 (64.3%)	130 (68.4%)
*Cessation before pregnancy*	123 (26.1%)	94 (25.3%)	59 (26.3%)	45 (23.7%)
*Smoked during pregnancy*	45 (9.6%)	37 (10.0%)	21 (9.4%)	15 (7.9%)
Alcohol consumption				
*None*	407 (86.4%)	322 (86.8%)	192 (85.7%)	169 (89.0%)
*Yes*^*a*^	64 (13.6%)	49 (13.2%)	32 (14.3%)	21 (11.0%)
Maternal education^b^				
*Low*	61 (13.0%)	52 (14.0%)	31 (13.8%)	30 (15.8%)
*Middle*	157 (33.3%)	119 (32.1%)	77 (34.4%)	65 (34.2%)
*High*	253 (53.7%)	200 (53.9%)	116 (51.8%)	95 (50.0%)
**Newborn**				
Gestational age, weeks	39.9 (1.0)	39.9 (1.0)	40.0 (0.9)	39.9 (0.9)
Birth weight, g	3462 (424)	3455 (423)	3430 (427)	3481 (418)
Birth length, cm^c^	50.3 (1.9)	50.3 (1.9)	50.5 (1.9)	50.4 (1.9)
Sex				
*Male*	242 (51.4%)	192 (51.8%)	117 (52.2%)	104 (54.7%)
Ethnicity^d^				
*European*	411 (87.3%)	323 (87.1%)	192 (85.7%)	156 (82.1%)
*Non-European*	60 (12.7%)	48 (12.9%)	32 (14.2%)	34 (17.9%)
Parity				
*1*	249 (52.8%)	205 (55.3%)	110 (49.1%)	102 (53.7%)
*2*	159 (33.8%)	121 (32.6%)	79 (35.3%)	58 (30.5%)
*≥ 3*	63 (13.4%)	45 (12.1%)	35 (15.6%)	30 (15.8%)
Season at delivery				
*Winter (Dec 21 to March 20)*	109 (23.1%)	84 (22.6%)	49 (21.9%)	41 (21.6%)
*Spring (March 21 to June 20)*	112 (23.8%)	90 (24.3%)	55 (24.5%)	45 (23.7%)
*Summer (June 21 to Sept 22)*	136 (28.9%)	106 (28.6%)	60 (26.8%)	54 (28.4%)
*Autumn (Sept 23 to Dec 20)*	114 (24.2%)	91 (24.5%)	60 (26.8%)	50 (26.3%)
Placental iodine concentration, μg/kg	26.1 (4.3)	26.1 (4.1)	26.1 (4.5)	26.5 (4.6)
Cord plasma insulin, pmol/L	–	36.2 (31.2)	–	–
Maternal plasma insulin, pmol/L	–	107.2 (188.4)	–	–
Cord plasma glucose, mmol/L	–	4.5 (1.1)	–	–
Maternal plasma glucose, mmol/L	–	4.6 (1.5)	–	–
HOMA-IR index	–	–	1.0 (0.7)	1.5 (1.6)
β-cell activity, %	–	–	106.6 (52.0)	134.8 (65.2)
Urinary iodine concentration, μg/L^e^	69.8 (58.3)			

Data are mean (SD) or *n* (%)

^a^Mothers who consumed a maximum of two glasses of alcoholic beverages per week

^b^Maternal education was coded as low (no diploma or primary school), middle (high school), and high (college or university degree)

^c^Data available for 468, 368, 224, and 189 participants, respectively

^d^Classification of ethnicity is based on the native country of the neonates’ grandparents as either European (at least two grandparents were European) or non-European (at least three grandparents were of non-European origin)

^e^Concentrations were measured for 98 individuals, and were reported previously [14]

### Placental iodine concentrations and GDM

**Fig. 2:**
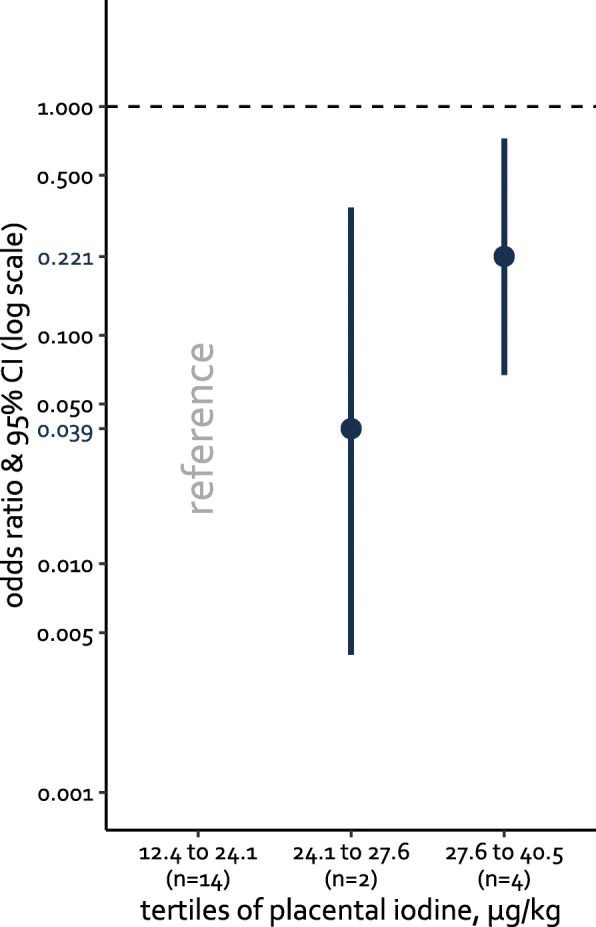
The placental iodine concentrations ranged from 12.4 to 24.1 μg/kg for the lowest tertile (*n* = 158), 24.1 to 27.6 μg/kg for the middle tertile (*n* = 157), and 27.6 to 40.5 μg/kg for the highest tertile (*n* = 156). *n* presents the number of women diagnosed with GDM in each tertile. Most mothers with GDM belonged to the lowest tertile (*n* = 14). Only two and four mothers with GDM were in the middle and highest tertiles, respectively. The odds ratio with 95% confidence intervals (95%CI) is presented on a log scale. Estimates and 95%CI for the middle and highest tertiles were calculated by contrasting them with the lowest tertile. Corrections were made for maternal education, hypertension, smoking status, pre-pregnancy BMI, maternal age at delivery, gestational weight gain, alcohol use, neonatal parity, gestational age, ethnicity, and sex of the neonate. On the *y*-axis, in red, the estimates are given

After correction for maternal education, hypertension, smoking status, pre-pregnancy BMI, age at delivery, gestational weight gain, alcohol use, neonatal parity, gestational age, ethnicity, and neonates’ sex, we observed in the total population that the likelihood of GDM decreased (OR = 0.82; 95%CI 0.72 to 0.93; *p* value = 0.003) with increasing placental iodine concentration. In Fig. 2, we contrasted the lowest tertile (i.e., 12.4 to 24.1 μg/kg) of placental iodine (*n* = 158) with the middle tertile (i.e., 24.1 to 27.6 μg/kg; *n* = 157) and the highest tertile (i.e., 27.6 to 40.5 μg/kg; *n* = 156). Compared to the lowest tertile, the odds at GDM were significantly lower in the middle tertile (OR = 0.039; 95%CI 0.004 to 0.36) and the highest tertile (OR = 0.22; 95%CI 0.067 to 0.72).

A total incidence of 20 GDM cases showed, on the basis of placental iodine tertiles, a PAF of 0.4 for the total mother population, indicating that 40% of the GDM is linked to lower placental iodine. Contrasting the lowest tertile of placental iodine with the highest tertile showed an estimated PAF of 54.2% (95%CI 11.4 to 82.3%; *p* = 0.001) of the GDM that could be prevented if all the mothers of the lowest placental iodine tertile (I < 24.1 μg/kg) would have the placental iodine level as the highest tertile (I ≥ 27.6 μg/kg).

### Placental iodine and plasma insulin concentrations

**Fig. 3:**
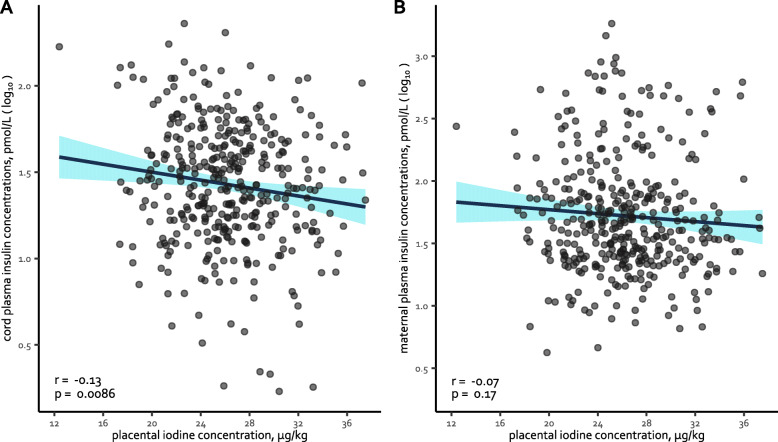
Correlation between cord (**a**) and maternal (**b**) plasma insulin and the placental iodine concentration (*n* = 371). Cord blood was collected at delivery, while maternal blood was sampled one day after delivery. Pearson’s correlation (*r*) was calculated and the corresponding *p* value is presented

The plasma insulin concentrations for 371 cord and maternal blood samples were available. Pearson’s correlations between the plasma insulin concentrations and the placental iodine levels were calculated and are presented in Fig. 3. In a multivariate analysis, for an increase in 5 μg/kg of placental iodine concentration, the insulin concentration in cord blood significantly decreased with 5.0% (95%CI − 9.1 to − 0.7%; *p* = 0.022). No significant association was observed between placental iodine and maternal insulin concentrations (*p* = 0.34).

### Placental iodine concentrations and HOMA-IR

The HOMA calculator was used to assess the degree of insulin resistance and β-cell activity [18]. The HOMA-IR index was log-transformed to normalize the distribution. The cord plasma HOMA-IR index was associated with a borderline significant decrease of 7.1% (95%CI − 14.1% to 0.5%; *p* = 0.067) for a 5 μg/kg increment in placental iodine load, while the same increment is associated with a lower β-cell activity (estimate = − 6.7%; 95%CI − 14.4 to 1.1%; *p* = 0.09). The HOMA-IR index for maternal plasma decreased with 11.3% (95%CI − 22.8 to 1.9%; p = 0.09) and the β-cell activity with 10.9% (95%CI − 21.8 to 0.0%; *p* = 0.05) for a 5-μg/kg increase in placental iodine.

### Sensitivity analyses

Exclusion of smokers did not alter the previously reported findings, nor did the addition of women with GDM to the models (data not shown).

## Discussion

Iodine deficiencies have been linked with a multitude of adverse health outcomes such as lower IQ points in children [21], attention deficit and hyperactivity disorders [22], and hypothyroidism [10]. The current study adds some key findings to this list: (1) a lower placental iodine load was linked with a higher risk at GDM, and, assuming for causality, over half of the GDM incidence could have been prevented if placental iodine levels of women from the lowest tertile would have been that of the highest tertile; (2) in neonates, the insulin concentration in cord plasma was inversely associated with placental iodine load; and 3) in both maternal and cord plasma, the HOMA-IR index and β-cell activity were negatively associated with placental iodine load, albeit with a borderline statistical significance.

In the literature on the risk at GDM, the relative importance of iodine sufficiency is still debated. A recent longitudinal case-control study investigated the association of FT_3_, FT_4_, and the FT_3_/FT_4_ ratio with GDM. The authors observed that FT_4_ concentrations were significantly lower in GDM cases in the first and second trimesters compared to non-GDM controls. At the same time, FT_3_ and the FT_3_/FT_4_ ratio was higher in women with GDM. Another prospective cohort of 27,513 pregnant women showed that lower levels of free thyroxine in early pregnancy (i.e., 9 to 14 weeks) were a risk factor for GDM incidence, as assessed at weeks 24 to 28 of gestation [7].

In contrast, a Finnish birth cohort found that lower maternal plasma iodine levels in the first trimester of pregnancy were not associated with risk at GDM [23]. However, it should be noted that Bell and colleagues [23] measured iodine in blood plasma, which reflects only the circulating levels of iodine and would, therefore, be a better proxy of thyroid function than iodine status [24, 25]. Their findings are in contrast to the placental iodine load, which mainly reflects the long-term gestational iodine status of women and hence would be more representative for the iodine status among pregnant women [14, 15]. Assuming for causality, our findings provide evidence of a higher incidence of GDM when placental iodine concentrations were in the lower range (PAF = 0.4). This is in agreement with observations made by Charoenratana et al. [26], as they observed that women in Thailand who were deficient in iodine (measured in urine) during pregnancy had a higher prevalence of GDM compared to iodine-replete women. Interestingly, the Belgian population [13] aligns more with the latter study in terms of gestational iodine deficiency, compared to the Finnish people,  whom have lower rates of gestational iodine deficiencies [27]. Although the current evidence seems inconclusive, our findings highlight a possible link between GDM and long-term gestational iodine deficiency.

It is thus plausible that a functional link exists between the iodine status and the onset of diabetes. This link could be traced back to insulin, as it recently became apparent that iodine deficiencies and insulin resistance are tightly related [28]. Several studies pointed out that urinary iodine concentrations were lower in people diagnosed with T2DM compared to the control groups and that the urinary iodine concentrations were inversely linked with the plasma insulin levels and the HOMA-IR index [11, 28, 29]. Although these studies concerning T2DM, GDM and T2DM share a similar pathophysiology: insulin resistance accompanied by a pancreatic β-cell insufficiency [30]. Along similar lines, we showed that placental iodine inversely associated with the insulin concentrations, the HOMA-IR index, and the β-cell activity at birth. We could still postulate that a mild-to-moderate iodine deficiency is linked with subclinical and early-onset changes in the insulin homeostasis in healthy pregnant women. Recently, it has been suggested that inflammatory cytokines, secreted by adipose tissue of insulin-resistant patients, and hyperinsulinemia itself could negatively modulate the expression of the sodium/iodide symporter on the apical surface of enterocytes, thus inducing a decrease in iodine absorption [28, 31]. This implies a functional association (possibly a negative feedback loop) between insulin resistance and iodine deficiency.

Although the exact mechanism is still unclear, it could be postulated that iodine deficiencies lead to an altered thyroid hormone homeostasis and a dysregulation of glucose metabolism [7]. In rodent studies, Kemp and colleagues [12] observed that the glucose transporter 2 expression in the rats' liver, which plays a significant role in maintaining average blood glucose concentrations, was influenced by the thyroid status. Hence, it is possible that an altered thyroid status contributes to an altered hepatic glucose turnover [12]. Another postulated mechanism is that during hyperthyroidism, the half-life of insulin is reduced, the degradation of insulin is heightened, and biologically inactive proinsulin is released [32]. Contrastingly, during hypothyroidism, liver glucose production is decreased [32], which is in line with Kemp et al. [12]. This would lead to lower insulin requirements in diabetic, hypothyroid patients. Another potential mechanism could be investigated via the triage theory of Bruce Ames [33]. According to this theory, episodic shortages in micronutrients are handled in a triage-like allocation mechanism, which prefers short-term “survival” over long-term adverse health effects. Because iodine is involved in various pathways, it is possible that the allocation mechanism could prefer the use of iodine in only a select number of structurally important pathways. For example, the use of iodine in the thyroid hormone production could be a preferred pathway over the use of iodine as an antioxidant agent. Iodine deficiencies have been linked to increased amounts of oxidative stress and a lower antioxidant capacity in pregnant women [34]. Interestingly, women with GDM had a higher presence of oxidative stress in the placenta compared to healthy controls [35]. It could prove informative to investigate a broader range of iodine-associated pathways at the same time in order to examine the effect of mild-to-moderate iodine deficiency thoroughly.

### Strengths and limitations

Our study has several strengths: Firstly, compared to other studies that used urinary iodine concentrations to estimate gestational iodine status, we used the placental iodine load as a marker for long-term gestational iodine accumulation. This biomarker proves to be more reliable and accurate compared to the short-term feature of urinary iodine concentrations [14, 15]. Secondly, the diagnosis of GDM was assessed by using the clinical biomarkers (i.e. GCT and OGTT) and performed by physicians from the recruitment hospital according to the American Diabetes Association guidelines [17]. Thirdly, our findings are generalizable to the Belgian gestational population (Additional file 2: Table S2).

We acknowledge some potential limitations. Because we used placental iodine concentrations of term-born children, no dynamic time-dependent iodine concentrations could be determined. The number of women diagnosed with GDM in the current study is low (*n* = 20; 4.3%). Nevertheless, this incidence of GDM is generally found for normal populations [2]. Moreover, our PAF calculation estimated that over 50% of the GDM cases could be prevented, which is in line with findings from a meta-analysis by Toulis and colleagues [8], who provided evidence that the risk at GDM increased by 50% in women with subclinical hypothyroidism. Given the observational study design, we cannot infer a causal link between placental iodine load and GDM. Even though previous findings indicated that an altered thyroid hormone status is more likely to cause GDM, it is still possible for GDM to affect thyroid hormones. For example, in diabetic patients, the thyroid-stimulating hormone response to the thyrotropin-releasing hormone is impaired [32]. Moreover, the peripheral conversion from thyroxine to triiodothyronine in diabetics could be impaired as well [32].

## Conclusions

Gestational iodine intake is essential as it provides a healthy intrauterine environment for optimal fetal growth. The current study sheds some light on the link between placental iodine load and the heightened risk at GDM, as well as an association with altered plasma insulin concentration, HOMA-IR index, and β-cell activity. The specific mechanisms of how the iodine status, prior to, and during gestation could heighten the risk at GDM remains to be unraveled. Nevertheless, our findings on GDM and placental iodine may help to improve the understanding of the developmental origins of health and disease. Our study coveys an important public health message, in that maintaining an adequate iodine status in the population at large may avert a significant portion of gestational diabetes mellitus and adverse health effects later in the neonates’ life.

Clinicians and healthcare managers should keep in mind that combatting iodine deficiency is complex and needs careful planning, even before pregnancy. Current evidence suggests that starting iodine supplementation during gestation might not be the most effective method to optimize the iodine supply to the mother and fetus [36].

## Supplementary Information

**Additional file 1: Table S1.** Maternal and neonate characteristics of 1) the original population of 794 mother-neonate pairs who were enrolled in the ENVIR*ON*AGE birth cohort between March 1^st^, 2013 and April 1^st^, 2017; 2) The randomly selected population (*n* = 498) for which we had placental iodine concentrations; and 3) the final study population (*n* = 471) after exclusion of preterm births, pre-eclamptic pregnancies, and mothers with thyroid problems.

**Additional file 2: Table S2.** Maternal and neonate characteristics of the current study group (n = 471) compared with a reference population of births in Flanders, Belgium (born 2002 until 2011; *n* = 606,877).

## Data Availability

The datasets used and/or analyzed during the current study are available from the corresponding author on reasonable request.

## Abbreviations

CI: Confidence intervals
ENVIR*ON*AGE: Environmental Influences ON Early Ageing
GDM: Gestational diabetes mellitus
OR: Odds ratio
PAF: Population attributable factor
SHS: Second-hand smoke
SD: Standard deviation
T2DM: Type 2 diabetes mellitus
TMAH: Tetramethylammonium hydroxide
