# Supplementary material for: Lower iodine storage in the placenta is associated with gestational diabetes mellitus
Source: BMC Med. 2021 Feb 19;19:47. doi: 10.1186/s12916-021-01919-4 (PMC7893873; doi:10.1186/s12916-021-01919-4)
Supplement: Supplementary file 1 — Additional file 1: Table S1. Maternal and neonate characteristics of 1) the original population of 794 mother-neonate pairs who were enrolled in the ENVIRONAGE birth cohort between March 1st, 2013 and April 1st, 2017; 2) The randomly selected population (n = 498) for which we had placental iodine concentrations; and 3) the final study population (n = 471) after exclusion of preterm births, pre-eclamptic pregnancies, and mothers with thyroid problems. [file 12916_2021_1919_MOESM1_ESM.docx]

**Additional File 1: Table S1: Maternal and neonate characteristics** **of 1) the original population of 794 mother-neonate pairs who were enrolled in the ENVIR*ON*AGE birth cohort between March 1^st^, 2013 and April 1^st^, 2017; 2) The randomly selected population (n=498) for which we had placental iodine concentrations; and 3) the final study population (n=471) after exclusion of preterm births, pre-eclamptic pregnancies, and mothers with thyroid problems.**

| **Characteristics** | **Original population (n=794)** | **Selected population (n=498)** | **Study population (n=471)** |
| --- | --- | --- | --- |
| **Mother** |  |  |  |
| Age, years | 29.7 (4.5) | 29.5 (4.4) | 29.4 (4.4) |
| Pre-pregnancy BMI, kg/m^2^ | 24.7 (4.9) | 24.6 (4.8) | 24.6 (4.8) |
| Net weight gain, kg | 13.9 (5.9) | 13.8 (5.8) | 13.9 (5.8) |
| Gestational diabetes mellitus |  |  |  |
| *No* | 755 (95.1%) | 477 (95.8%) | 451 (95.7%) |
| *Yes* | 39 (4.9%) | 21 (4.2%) | 20 (4.3%) |
| Hypertension |  |  |  |
| *No* | 754 (95.0%) | 470 (94.4%) | 443 (94.1%) |
| *Yes* | 40 (5.0%) | 28 (5.6%) | 28 (5.9%) |
| Self-reported tobacco use |  |  |  |
| *Non-smoker* | 514 (64.7%) | 321 (64.5%) | 303 (64.3%) |
| *Cessation before pregnancy* | 198 (24.9%) | 130 (26.1%) | 123 (26.1%) |
| *Smoked during pregnancy* | 82 (10.4%) | 47 (9.4%) | 45 (9.6%) |
| Alcohol consumption |  |  |  |
| *None* | 687 (86.6%) | 432 (86.8%) | 407 (86.4%) |
| *Yes^a^* | 107 (13.4%) | 66 (13.2%) | 64 (13.6%) |
| Maternal education^b^ |  |  |  |
| *Low* | 90 (11.3%) | 64 (12.9%) | 61 (13.0%) |
| *Middle* | 293 (36.9%) | 163 (32.7%) | 157 (33.3%) |
| *High* | 411 (51.8%) | 271 (54.4%) | 253 (53.7%) |
| **Newborn** |  |  |  |
| Gestational age, weeks | 39.8 (1.2) | 39.8 (1.1) | 39.9 (1.0) |
| Birth weight, g | 3,431 (463) | 3,452 (436) | 3,462 (424) |
| Birth length, cm^c^ | 50.3 (2.0) | 50.3 (1.9) | 50.3 (1.9) |
| Sex |  |  |  |
| *Male* | 415 (52.3%) | 256 (51.4%) | 242 (51.4%) |
| Ethnicity^d^ |  |  |  |
| *European* | 689 (86.8%) | 437 (87.8%) | 411 (87.3%) |
| *Non-European* | 105 (13.2%) | 61 (12.2%) | 60 (12.7%) |
| Parity |  |  |  |
| *1* | 399 (50.3%) | 261 (52.4%) | 249 (52.8%) |
| *2* | 292 (36.8%) | 172 (34.5%) | 159 (33.8%) |
| *≥ 3* | 103 (13.0%) | 65 (13.1%) | 63 (13.4%) |
| Season at delivery |  |  |  |
| *Winter (Dec 21 to March 20)* | 196 (24.7%) | 117 (23.5%) | 109 (23.1%) |
| *Spring (March 21 to June 20)* | 181 (22.8%) | 116 (23.3%) | 112 (23.8%) |
| *Summer (June 21 to Sept 22)* | 204 (25.7%) | 139 (27.9%) | 136 (28.9%) |
| *Autumn (Sept 23 to Dec 20)* | 213 (26.8%) | 126 (25.3%) | 114 (24.2%) |

*Data are mean (SD) or n (%).
^a^ Mothers who consumed a maximum of two glasses of alcoholic beverages per week. ^b^ Maternal education was coded as low (no diploma or primary school), middle (high school), and high (college or university degree).
^c^ Data available for 788, 495, and 468 participants respectively.
^d^ Classification of ethnicity is based on the native country of the neonates' grandparents as either European (at least two grandparents were European) or non-European (at least three grandparents were of non-European origin).*
