# Supplementary material for: Lower iodine storage in the placenta is associated with gestational diabetes mellitus
Source: BMC Med. 2021 Feb 19;19:47. doi: 10.1186/s12916-021-01919-4 (PMC7893873; doi:10.1186/s12916-021-01919-4)
Supplement: Supplementary file 2 — Additional file 2: Table S2. Maternal and neonate characteristics of the current study group (n = 471) compared with a reference population of births in Flanders, Belgium (born 2002 until 2011; n = 606,877). [file 12916_2021_1919_MOESM2_ESM.docx]

**Additional File 2: Table S2: Maternal and neonate characteristics** **of the current study group (n=471) compared with a reference population of births in Flanders, Belgium (born 2002 until 2011; n=606,877).**

| **Characteristics** | **Total population (n=471)** | **Births in Flanders (n=606,877)^a^** |
| --- | --- | --- |
| **Maternal** |  |  |
| Age, years | 29.4 (24.0 – 35.0) | 29.5 (23.5 – 35.8) |
| < 25 | 77 (16.3%) | 98,419 (16.2%) |
| 25 – 34 | 335 (71.1%) | 428,781 (70.7%) |
| 35+ | 59 (12.6%) | 79,677 (13.1%) |
| Maternal education^b^ |  |  |
| *Low* | 61 (13.0%) | 58,743 (13.1%) |
| *Middle* | 157 (33.3%) | 183,410 (40.8%) |
| *High* | 253 (53.7%) | 207,563 (46.2%) |
| **Newborn** |  |  |
| Birth weight, g | 3,462 (2,875 – 4,015) | 3,360 (2,740 – 3,965) |
| Sex |  |  |
| *Male* | 242 (51.4%) | 311,620 (51.4%) |
| Ethnicity^c^ |  |  |
| *European* | 411 (87.3%) | 384,522 (87.7%) |
| Parity |  |  |
| *1* | 249 (52.8%) | 284,770 (46.9%) |
| *2* | 159 (33.8%) | 210,731 (34.7%) |
| *≥ 3* | 63 (13.4%) | 111,376 (18.4%) |
| Month of birth |  |  |
| *January* | 42 (8.9%) | 50,929 (8.4%) |
| *February* | 31 (6.6%) | 46,524 (7.7%) |
| *March* | 31 (6.6%) | 51,541 (8.5%) |
| *April* | 25 (5.3%) | 50,035 (8.2%) |
| *May* | 42 (8.9%) | 50,771 (8.4%) |
| *June* | 53 (11.3%) | 50,975 (8.4%) |
| *July* | 40 (8.5%) | 53,436 (8.8%) |
| *August* | 49 (10.4%) | 53,366 (8.8%) |
| *September* | 40 (8.5%) | 51,702 (8.5%) |
| *October* | 38 (8.1%) | 51,397 (8.5%) |
| *November* | 42 (8.9%) | 47,033 (7.8%) |
| *December* | 38 (8.0%) | 49,168 (8.1%) |

*Data are presented as median (10^th^ percentile – 90^th^ percentile) for continuous variables, and as n (%) for discrete variables.*

*^a^Cox, B., E. Martens, B. Nemery, J. Vangronsveld and T. S. Nawrot (2013). "Impact of a stepwise introduction of smoke-free legislation on the rate of preterm births: analysis of routinely collected birth data." BMJ 346: f441.*

*^b^ Coded as ‘low’ (no diploma or primary school), ‘middle’ (high school) or ‘high’ (college or university degree).*

*^c^ Classification is based on the native country of the neonates' grandparents as either European (at least two grandparents were European) or non-European (at least three grandparents were of non-European origin.*
